# A Large Follicular Adenomatoid Odontogenic Tumor Occupying the Maxillary Sinus: A Case Report

**DOI:** 10.31729/jnma.8071

**Published:** 2023-03-31

**Authors:** Leison Maharjan, Urmila Gurung, Bibhu Pradhan

**Affiliations:** 1 Department of Otolaryngology and Head and Neck Surgery, Patan Academy of Health Sciences, Lagankhel, Lalitpur; 2 Nepal, Department of ENT-Head and Neck Surgery, Tribhuvan University Teaching Hospital, Maharajgunj, Kathmandu, Nepal

**Keywords:** *adenomatoid tumor*, *case reports*, *hamartoma*, *odontogenic cysts*

## Abstract

Adenomatoid odontogenic tumour is a rare benign, odontogenic tumour with uncertain histogenesis. Whether it is a hamartoma or a neoplasm is still a controversial topic. It is usually associated with an unerupted maxillary canine. Here, we discuss a follicular adenomatoid odontogenic tumour in a young girl with uncommon features such as it arose from two unerupted teeth and partial resorption of the roots of other normal teeth. The tumour was large enough to completely occupy the maxillary sinus. It was treated with enucleation and curettage by lateral rhinotomy approach.

## INTRODUCTION

Adenomatoid odontogenic tumour (AOT) is a unique rare odontogenic tumour usually associated with an unerupted maxillary cuspid in teenage girls whose incidence ranges from 2.2% among 230 odontogenic tumours in a study done in Central Europe upto 7.1% among 34 odontogenic tumours in a study done in Mexico.^1,2^ Though many terms have been used to refer to this tumour in history, the term AOT was adopted in the initial edition of WHO's Histological Typing of Odontogenic Tumors, Jaw Cysts and Allied Lesions in 1971. It was retained in the second edition.^1,3^ Whether AOT is a hamartoma or a neoplasm is still controversial.^1^ Here we discuss such unusual AOT in a young girl who was managed by excision and curettage by lateral rhinotomy approach.

## CASE REPORT

**Figure 1:**
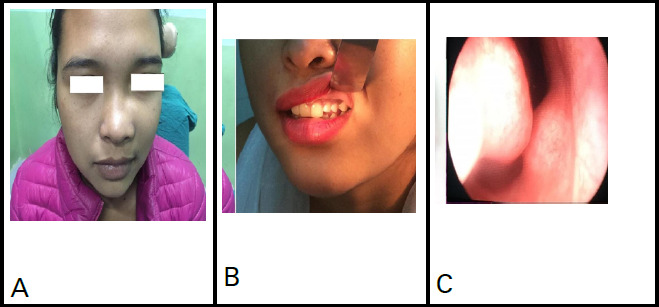
A) Swelling in the left maxillary region obliterating the left nasofacial groove, B) Deciduous teeth-upper left lateral incisor and upper left canine, C) Swelling in the floor of the right nasal cavity due to an unerupted canine.

**Figure 2:**
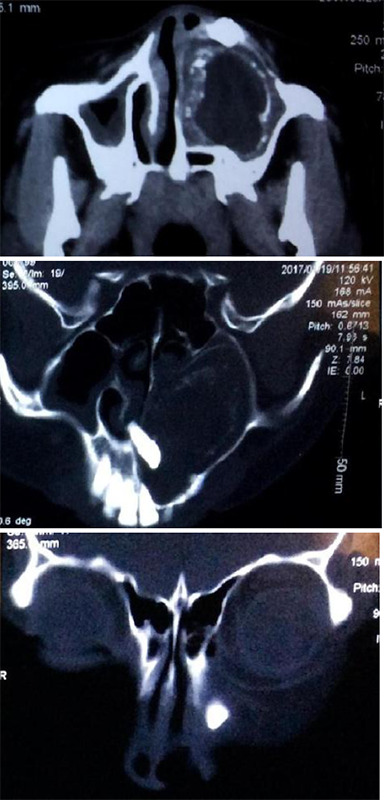
CECT showing heterogeneously hypodense lesion occupying the left maxillary sinus with thinned-out walls and signs of bony erosion. Coronal cuts showing the unerupted left upper canine and left upper lateral incisor.

A 16-year-old girl presented with a gradually progressive swelling in the left cheek for 9 months causing left nasal obstruction and epiphora. She also complained of swelling in the hard palate on the same side for 3 months. On clinical examination, there was a firm, smooth swelling in the left maxillary region obstructing the left nasal cavity completely, pushing the nasal septum to the other side. The same swelling expanded through the hard palate into the oral cavity obliterating the left gingivolabial sulcus. She had two deciduous teeth-upper left lateral incisor and upper left canine. Two unerupted teeth, an incisor could be felt just inferior to the left medial canthus and a canine could be felt in the floor of the right nasal cavity (Figure 1, 2).

Contrast enhanced computed tomography (CECT) revealed a heterogeneously hypodense lesion measuring 4.5x3.5x3.2 cm arising from the alveolar process of the maxilla on the left side completely expanding into the left maxillary sinus. All the walls of the sinus were thinned out without evidence of any local invasion. Two impacted teeth were seen at the anteroinferior and anterosuperior aspects of the lesion. No significant enhancement in the central component of the lesion was noted (Figure 2). Fine needle aspiration reported the lesion to be an odontogenic neoplasm.

**Figure 3:**
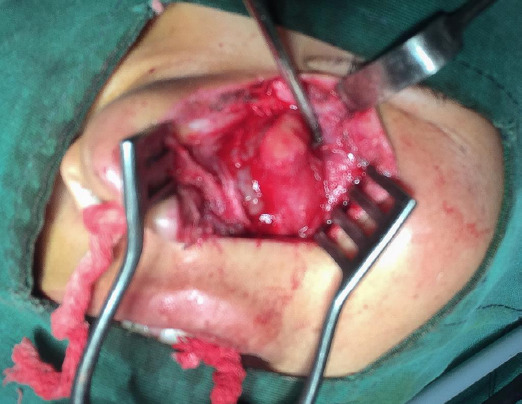
AOT as seen during the lateral rhinotomy approach.

**Figure 4:**
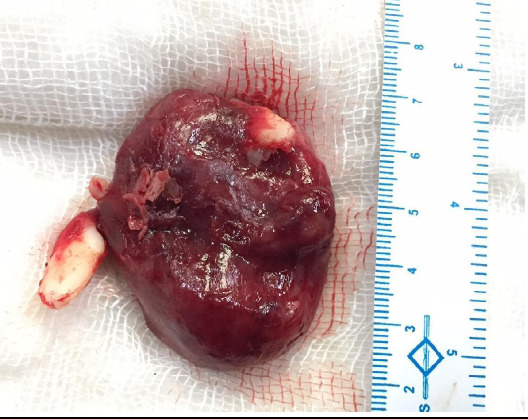
A thick-walled cystic mass covering the entire left maxillary sinus with unerupted left canine attached to inferomedial portion of AOT and left upper lateral incisor was attached to the superior surface of the cyst.

The patient underwent enucleation and curettage via lateral rhinotomy approach under general anaesthesia (Figure 3). Macroscopically, the tumour was a thick, smooth-walled, cystic mass containing a brownish fluid (Figure 4).

**Figure 5:**
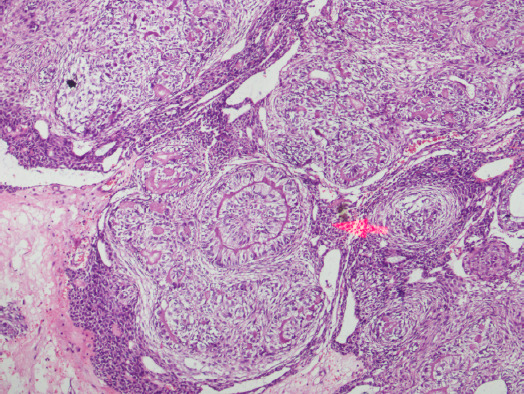
Duct-like structures, convoluted structures, with some ducts containing amorphous eosinophilic material (arrow) (Hematoxylin-eosin stain, original magnification x 20).

The unerupted left canine was situated in the inferomedial portion of tumor and left upper lateral incisor was situated on the superior surface. Inferiorly, the tumour extended upto the alveolus and hard palate, partial resorption of the roots of the upper teeth. Microscopically, the tissue showed duct-like and convulated structures, with some ducts containing amorphous eosinophilic material. With these features the histopathological diagnosis of AOT was made (Figure 5). We followed up our case upto 1 year without any recurrence.

## DISCUSSION

The AOT is an uncommon benign odontogenic tumour with slow progressive growth. This tumour is also referred to as a "two-thirds tumour" because about two-third cases arise in young females, two-thirds cases occur in the maxilla, two-third cases are associated with an unerupted tooth, and two-third affected teeth are canines.^4^ The histologic typing of the WHO defined AOT as a tumour composed of odontogenic epithelium presenting a variety of histoarchitectural patterns, embedded in a mature connective tissue stroma, and characterized by slow but progressive growth.^5^

There are major two forms of AOT-intraosseous (central) and extraosseous (peripheral). The intraosseous form has two types- a follicular (or peri coronal) and an extrafollicular (or extra-coronal) type. Radiographically, the follicular type can be seen as a well-defined, unilocular round or ovoid-shaped, radiolucent lesion. It often mimics a dentigerous or follicular cyst as it is associated with the crown and often part of the root of an unerupted tooth. Whereas the extrafollicular type is not associated with an unerupted tooth and the well-defined, unilocular radiolucency is found between, above, or superimposed upon the roots of the erupted, permanent tooth. In two-thirds of the intraosseous form, the radiolucency shows discrete foci having a flocculent pattern of scattered radiopacities. On the other hand, the peripheral form of AOT appears as a gingival fibroma or epulis attached to the labial gingiva. In this type, slight erosion of the alveolar bone crest is seen but radiographic changes are not that prominent.^2^ In our case, the tumor was a follicular intraosseous type with features similar to those described above.

Females are affected by AOT more than males with a female: male ratio of 1.9:1. It is even marked in Asians, with a ratio of female: male 3:1.^2,6^ It was found that the lesion was common in the second decade of life with a mean age of 19 years old.^6^ The sex and the age of the patient we described, in this case, are consistent with the literature. Usually, most of the patients with AOT are asymptomatic and discovered on routine dental radiographic examination.^1^ Similar to our case, the most common symptoms that the patients present with are intraoral and extraoral painless slow-growing swelling.^6^

Our case has a few unusual features. Firstly, due to the position of the tumor, our patient presented with rare symptoms of nasal obstruction and epiphora. Secondly, most AOTs are between 1 cm and 3 cm,^1^ but in our case tumor was large measuring 4.5 cm in greatest diameter, and occupied the maxillary sinus completely. It also caused slight facial asymmetry. Only a few large cases have been reported.^7,8^ Thirdly, usually, such tumors are associated with only one impacted tooth,^1^ and only a few have been reported with multiple impacted teeth,^9^ whereas in our case it was associated with two unerupted teeth. Lastly, there was resorption of the roots of the few upper teeth which is an uncommon feature in AOT.^10^

Due to uncertain histogenesis, sometimes AOT is categorized as a hamartomatous lesion, and sometimes as a neoplasm.^1,10^ The large tumor causing facial asymmetry in our patient favors AOT to be a true neoplasm as debated in one of the cases reports.^10^

Radiologically in most of cases, AOT appears as a well-demarcated, unilocular radiolucent lesion, and may contain fine calcifications.^1,6^ Unerupted teeth were associated with the tumor in 73% of the cases majority of which were permanent teeth.^6^ The findings are consistent with our case. Radiologically differential diagnosis of follicular AOT includes radiolucent lesions like a dentigerous cyst, odontogenic keratocyst, calcifying odontogenic cyst, unicystic ameloblastoma, ameloblastic fibroma, early ameloblastic fibro-odontoma, odontogenic fibroma, and calcifying epithelial odontogenic tumor.^1^

Histologically, AOT exhibits diverse histomorphic features but the light microscopic findings are unique and consistent. Usually, tumor has a fibrous capsule of variable thickness. Although present in various proportions, the tumor is made up of cellular multinodular proliferation of spindle, cuboidal and columnar cells in a variety of patterns, usually scattered in duct-like and rosettes structures and calcifications in several forms.^1,2,6,10^ Eosinophilic amorphous homogenous material (tumour droplets) is usually seen in the core of the rosettes.^2,6,11^ Furthermore, darkly staining dystrophic calcifications in inconsistent amounts can also be seen in these lesions. Other materials which are associated with degraded enamel such as hyaline, dysplastic or calcified osteodentin, are other uncommon findings in AOTs.^5^ Usually histochemical, ultrastructural findings, and immunohistochemical analyses are not needed to diagnose AOT but these have been used for research purposes to determine the cell of origin.^1^ Treatment is usually by conservative surgical enucleation or curettage. Recurrence is very rare.^1,6^ We followed up our case for upto one year without any recurrence.

Adenomatoid odontogenic tumor (AOT) is a rare odontogenic tumor. It should be considered as a differential diagnosis in radiolucent maxillary swelling with unerupted teeth. It can be managed by enucleation and curettage with excellent results.
